# Supplementary material for: Oxazepam and cognitive reappraisal: A randomised experiment
Source: PLoS One. 2021 Apr 22;16(4):e0249065. doi: 10.1371/journal.pone.0249065 (PMC8061924; doi:10.1371/journal.pone.0249065)
Supplement: Attachment — Submitted filename: response_to_reviewers.docx [file pone.0249065.s001.docx]

***Response to reviewers***

*We thank the reviewers and the editor for the valuable comments, which have improved the manuscript. Responses to the points are provided below in* ***bold****. Changes in the manuscript are highlighted.*

[…] given the small and male-only sample, the study should be handled as a pilot study. Could you please designate the study as a pilot study in the abstract.

**We fully agree with the comment that the relatively small, male-only sample has limited generalizability. In keeping with the spirit of this request, we now designate the study as a preliminary investigation in the abstract, since the study was not designed specifically as a pilot study (i.e. it was designed for hypothesis testing, albeit of a preliminary nature, rather than for evaluating feasibility of the protocol).**

2. We noticed you have some minor occurrence of overlapping text with the following previous publication, which needs to be addressed: <https://royalsocietypublishing.org/doi/10.1098/rsos.160607>

The text that needs to be addressed involves lines 15-20 only. In your revision ensure you cite all your sources (including your own works), and quote or rephrase any duplicated text outside the methods section. Further consideration is dependent on these concerns being addressed.

**We have rephrased the sentences in question.**

3. Thank you for including your ethics statement:  "The study was approved by the regional ethical review board of Stockholm (no. 2009/1128-31/3). Participants gave written informed consent."

a) Please amend your current ethics statement to include the full name of the ethics committee/institutional review board(s) that approved your specific study.

b) Once you have amended this/these statement(s) in the Methods section of the manuscript, please add the same text to the “Ethics Statement” field of the submission form (via “Edit Submission”).

For additional information about PLOS ONE ethical requirements for human subjects research, please refer to http://journals.plos.org/plosone/s/submission-guidelines#loc-human-subjects-research."

**The full name of the ethical review board is the “regional ethical review board of Stockholm”, as stated. For reference, see e.g.** [**https://www.government.se/government-agencies/ethical-review-boards-etikprovningsnamnderna/**](https://www.government.se/government-agencies/ethical-review-boards-etikprovningsnamnderna/)**. (The ethical review boards in Sweden have since been reorganised into a single national authority.)**

***Comments to the Author***

Reviewer #1: This is an interesting study in which the effect of Oxazepam on cognitive reappraisal has been investigated in 20 healthy male subjects (HC) compared to 13 healthy male subjects who received placebo. For reappraisal IAPS pictures were used. Skin conductance, electromyographic (EMG) activity over the superciliary corrugator muscle, and heart rate were investigated during emotional up- and downregulation in response to negative and neutral IAPS pictures. The data are part of a larger study, thus a larger number of clinical rating scales were also applied (IRI, STAI, TAS-20, PPI-R).

The upregulation and downregulation of negative-valence images caused changes in unpleasantness ratings, corrugator activity, and heart rates in the expected direction which can be regarded as a confirmation of the effects of cognitive control strategies over emotional reactions. Patients with Oxazepam showed lower ratings of unpleasantness to negative stimuli, but oxazepam did not have any effect on reappraisal. Thus, cognitive emotion regulation is not suppressed by benzodiazepines. These data are very much in line with clinical observations which support the hypothesis of an effect of benzodiazepines on emotion perception related to the amygdala, but not on cognitive regulation strategies related to the prefrontal cortex.
Major limitation of the study is the small number of only male subjects.
Overall, these data can be regarded as pilot data presenting a valid paradigm for reappraisal of negative emotions which could be used in different neuropsychiatric disorders and with different pharmaceutical interventions.
Just one suggestion:
It would be of interest to include the results of the rating scales in another table to get a better idea of the variance in personality traits, as one suspects that, e.g. psychopathy, might have been overall very low in this group.

**We have added a table (table 1) showing participant characteristics and rating scale scores, as requested**

Reviewer #2: This paper addresses potential effects of the benzodiazepine oxazepam on cognitive reappraisal of emotional images. Two groups of participants (one given oxazepam, one given a placebo) attempted to up- and downregulate negative and neutral affective pictures, while various autonomic indices were recorded (skin conductance, heart rate, etc.). The research topic is certainly interesting and it seems the authors put great effort into data collection. I also appreciate the authors’ clearly communicating that this experiment is part of a larger study and them being straightforward about the problems they encountered during data collection (randomization error leading to loss of half their sample, data loss of the recall test that was supposed to ensure that participants adhered to reappraisal instructions). Yet, there are some major and minor issues that prevent me from recommending publication at this point.

\## Introduction:

#1 Major point(s): As it currently stands, the introduction does not provide enough background to understand the conducted experiment. While the authors provide some information on cognitive reappraisal and benzodiazepines, there is no relatable transition between these two paragraphs (p. 2, lines 15 ff.), making it hard to understand how they are related. I understand the general idea is that benzodiazepines, similar to reappraisal, reduce emotional responding to negative stimuli and hence it may be interesting to examine their interactions in this regard. Yet the authors do not elaborate on what these potential interactions may look like in detail and why they may occur. As a reader, I am left with several questions. For example, why are benzodiazepines expected to reduce the effects of cognitive reappraisal in particular? Due to their anxiety-relieving nature, should they not affect emotion regulation in general? Why is it interesting to look at the specific interactions with cognitive reappraisal? Only from the discussion do I finally get that there is a theory that benzodiazepines may suppress top-down regulatory mechanisms, however, this should already be explained in the introduction to establish the research questions. Furthermore, other emotion regulation strategies also depend on top-down control (e.g., attentional control). I think a clearer derivation of the study goal is needed here. In addition, I am faced with another question: Wouldn’t benzodiazepines naturally reduce participants’ efforts to engage in emotion regulation to begin with? If benzodiazepines reduce emotional responding, is there even a need for individuals to attempt emotion regulation in the first place? How would you make sure that benzodiazepines in fact reduce the ability for using cognitive reappraisal and do not simply reduce emotional responding in a way that renders reappraisal efforts unnecessary to the individual? Also, why was oxazepam chosen as the medication of interest? Here, I need more background and literature on effects of benzodiazepines or equivalent substances on emotional processing/regulation that make the purpose of this research more easily comprehensible.

**We agree with the reviewer that benzodiazepines potentially can affect different types of emotional regulation. Cognitive reappraisal is a well-studied emotional regulation strategy which also have clinical relevance. We have added a first paragraph introducing cognitive reappraisal as one strategy for emotional regulation.**

**We understand the reviewer’s comment that the intrinsic motivation to engage in emotional regulation would be expected to be lower if participants’ spontaneous emotional response was substantially reduced in first place. In this specific study, participants still reported negative affect in response to the stimuli suggesting that there would still be a “need” for emotional regulation.**

**Oxazepam is a benzodiazepine with a relatively strong anxiolytic effect and weaker hypnotic effect, why this medication was used. This has now been clarified in the introduction.**

**We have added a paragraph in the beginning of the introduction that introduces the research question and added references to some previous experimental work on bensodiazepines and emotional processing.**

#2 Related to this point, there are no clear research questions or hypotheses for all the dependent variables investigated (heart rate, skin conductance, corrugator muscle activity, ratings). In order to evaluate the statistical analyses and to reasonably interpret the data, the authors should inform readers about their specific expectations, e.g., was oxazepam expected to reduce or increase autonomic indices of arousal, for what reappraisal instruction, and why?

**We have rephrased the aims section to clarify hypotheses for the respective outcomes.**

#3 The sentence on criminal violent behavior comes out of nowhere and is confusing (p. 2, lines 24 ff.) How does this information relate to the rest of the introduction and the manuscript? Even if the authors’ idea of benzodiazepine suppressor effects on top-down control stems from the disinhibition theory of criminal behavior (p. 8, lines 280 ff.), I think this information is out of place in the introduction, as readers cannot relate to it.

**We have rewritten the introduction to clarify the reasoning with respect to this point.**

#4 Minor point: bensodiazepines should be benzodiazepines?

**We have corrected the spelling throughout.**

\## Methods:

#5 Major point: I understand that details of the recruiting process and experimental design have been published elsewhere (Nilsonne et al., 2017). I also understand that the reappraisal paradigm was adapted from Golkar et al. (2012). Nonetheless, information crucial to understanding the current experiment must also be mentioned in the current manuscript, as readers cannot be expected to switch to several other manuscripts for necessary details. This is most evident in the brief description of the reappraisal paradigm. First, there is no information on number of trials and duration of trials, which should be explicitly stated (providing this information in the figure only is not enough). Secondly, it is not clearly stated that participant reappraised negative and neutral pictures, which again only becomes clear when closely looking at Figure 1, and may thus easily confuse readers. Further, in the paper of Golkar et al. (2012), it reads that participants received an “attend” and a “reappraise” instruction (not an “upregulate” and “downregulate” instruction). Was an “attend” instruction also used in the present study? If not, merely referring readers to the paper of Golkar et al. (2012) adds to the confusion, if the paradigms are in fact not identical. It should also be clarified when participants rated the unpleasantness of stimuli (before or after reappraisal?). I am guessing before, but it needs to be stated more clearly. Altogether, a detailed description of the reappraisal paradigm should be added to this manuscript.

**We have added the requested information to the methods section and expanded the description of the paradigm.**

#6 The authors specifically mention anxiety regulation as a common denominator for cognitive reappraisal and benzodiazepines in the introduction, making me wonder about the utilized stimulus material in the experiment. I can guess from figure 1 that both neutral and negative affective images were used (please also see #5). Were the negative pictures specifically anxiety-eliciting? If no, what was the rationale for using negative pictures in general, if part of the introduction is specifically built on anxiety regulation?

**It is correct that neutral and negatively valenced IAPS images were used, and we have expanded the methods description on this point. This type of stimulus material is very well-established in experimental paradigms for cognitive reappraisal.**

#7 The authors administered a large variety of rating scales on personality, anxiety, etc. While I understand that the experiment was not designed for effects on cognitive reappraisal alone (but also emotional mimicry and empathy for pain), it would have been helpful to assess participants’ habitual reappraisal tendencies, reappraisal frequency or beliefs in reappraisal efficacy as well. While there is no obvious reason to expect that the two randomly created groups differ greatly in these parameters (as in one is more competent then the other), interactions may still emerge regarding the effects of oxazepam on reappraisal. E.g., it may be plausible that oxazepam only reduces reappraisal effects in individuals who are less familiar with this strategy, but not in individuals who use reappraisal regularly and thus are likely more capable to implement it under various circumstances. Or likewise, individuals who do not belief reappraisal to be an effective regulation strategy in the first place may be more susceptible to the effects of oxazepam on their reappraisal process. I think this should at least be discussed in the limitations.

**We have added the possibility of imbalance in baseline reappraisal capacity/tendency as a possible limitation, which cannot be excluded in a between-group randomised design.**

#8 Minor point: I recommend using a negative image in Figure 1 to better underline the purpose of the experiment (provided the snail is a neutral image).

**We have replaced the image of the snail with an image of a negative stimulus (not from the actual stimulus set).**

#9 Minor point: The sentence “rating scales in this study have been described in…” (p.4, line 26) should be omitted, since the necessary scales are briefly explained anyway. Instead, the authors could add: For more details on the used rating scales, please see ….

**We have rephrased accordingly.**

\## Results

#10 The participants information in the results should be integrated into the participants information in the methods section. This way, readers are already aware of the final sample size (33 instead of the initial 39) and group sizes.

**Following the suggestion of reviewer 1, we are adding table 1 with results from rating scales. This table will occur in the beginning of the results section. For this reason, we have decided to maintain the “participants” heading under results. We have however revised the text to avoid redundancy.**

#11 Major point: The naming of variables when the authors report their interactions thoroughly confuses me. Am I correct in the assumption that “upregulation” actually refers to the within factor of reappraisal instruction (upregulate vs. downregulate) and that “negative stimulus valence” refers to the within factor of picture valence (negative vs. neutral)? In the same manner, does “oxazepam treatment” actually mean the between factor treatment group (oxazepam vs. placebo)?. The way the variables are named, the authors give the impression that they only report results on upregulate instructions, negative valence stimuli, and participants receiving oxazepam. E.g., I initially had trouble figuring out where the downregulation condition went, before realizing there might be something wrong with the naming. Additionally, for better comprehensibility, I suggest adding to each interaction whether it is considered significant or not. Simply stating p-values unnecessarily hampers readability.

**The reviewer’s interpretation of the naming of variables is correct. We have revised the statistical methods to clarify the deviation coding scheme we have followed and its interpretation. We have also added interpretation of statistically significant effects in the results section.**

#12 Minor point: In the exploratory analyses, describing personality traits as predictors of cognitive reappraisal and then reporting effects on unpleasantness ratings is not correct, since unpleasantness ratings took place before cognitive reappraisal and are not part of the reappraisal process, but as the authors state, affective experience with the stimulus material.

**We have expanded the methods description to clarify this point.**

\## Discussion

#13 (also relates to the introduction). While it should have already been mentioned in the introduction, I am missing a critical discussion on the practical implications of the initial research question and the results. What is the relevance of this study in clinical settings where patients are given oxazepam for anxiety reduction? Initially, if interactions with reappraisal instructions were expected, as in that oxazepam could reduce the effects of cognitive reappraisal, would this suggest that administering oxazepam could diminish individuals’ natural reappraisal capacities? What would this mean in the long run? Given the clinical context that is build in the introduction, I need a more thorough discussion to understand the study rationale.

**We did not identify an effect of oxazepam on cognitive reappraisal, thus failing to corroborate the prediction that top-down regulatory mechanisms are suppressed by benzodiazepines, arising from the disinhibition theory of criminal violent behavior. The findings also do not suggest that benzodiazepines, although they have other risks in a clinical setting, contribute to poorer emotional regulation performance in patients. We have added this second point to the discussion.**
