# Supplementary material for: Oxazepam and cognitive reappraisal: A randomised experiment
Source: PLoS One. 2021 Apr 22;16(4):e0249065. doi: 10.1371/journal.pone.0249065 (PMC8061924; doi:10.1371/journal.pone.0249065)
Supplement: Attachment — Submitted filename: response_to_reviewers_2_2021-02-03.docx [file pone.0249065.s002.docx]

***Response to reviewers***

*We thank the reviewers and the editor for the valuable comments, which have improved the manuscript. Responses to the points are provided below in* ***bold****.*

Reviewer #1: The authors implemented most of my previous suggestions in this revision, which has improved the derivation of research questions, description of the study paradigm, and discussions of findings as well as limitations. A few minor issues need to be resolved before publication however.

General: In keeping with general terminology in the manuscript and to avoid confusion, “emotional reappraisal” should be changed to “cognitive reappraisal” throughout the manuscript (see abstract, conclusions, etc.)

**This has been changed throughout.**

Materials and Methods, participants: Since PLoS One is a multidisciplinary journal and not all readers may be familiar with substance interactions, please add a short statement as to why participants could not be habitual consumers of nicotine. Are there interactions of oxazepam to be expected?

**We excluded habitual consumers of nicotine to reduce the risk of abstinence symptoms during the experiment. We have now added this into the methods section (page 3, “Participants”).**

Results: The authors adopted the results section according to my previous comments (variable naming), but did not add information as to whether an effect is statistically significant or not. However, I am of the opinion that it would greatly increase comprehensibility on part of the readers to respectively state before reporting effects and p-values whether an effect/interaction was significant or not. Example for unpleasantness ratings: “The three-way interaction was not significant at -2.7….”. This should be changed for all effects and interactions before I recommend publication. Additionally, for skin conductance, main effect of up-regulation and corrugator activity, main effect of negative stimulus valence, the authors should give a short description of the effect instead of simply stating “… as expected”.

**This has been changed throughout.**

Associations between self-rated personality traits (p. 8): Please add that corresponding statistics can be found in Figure 9, as this is not immediately evident.

**This has been added (page 8, “Associations between self-rated personality traits to responses to stimuli and instructions to perform reappraisal”).**

Discussion:

p.9: the sentence “It has also been shown that these prefrontal processes interact with processing of emotional information in brain regions such as the amygdala” is missing references.

**We have added references as requested.**

p.9: the sentence “The findings also do not suggest that benzodiazepines […] contribute to poorer emotion regulation performance in patients” needs to be amended.
First, the authors did not directly assess emotion regulation performance, which would require objective analysis of number of reappraisal ideas and quality of ideas (see Papousek et al., 2017; Brain Imaging or Behavior, or Rowlands et al., 2019; Neuropsychological Rehabilitation). Much rather, reappraisal of IAPS pictures assesses individuals’ regulation success or reappraisal effectiveness in terms of differences in emotion intensity and physiological variables between two conditions (regulate up/down, etc.). Next, generalizing this study’s findings to “emotion regulation” in general does not seem justified, since only cognitive reappraisal was investigated. For these reasons, this sentence should read “The findings also do not suggest that benzodiazepines […] contribute to poorer cognitive reappraisal in patients.”

**This has been edited as suggested.**

Reviewer #2: Introduction
1. The authors should draw some stronger connections between the GABAa receptors and frontal lobe activity to help solidify the logic behind by cognitive reappraisal would be impacted by benzodiazepines. Within the final paragraph on page one, the authors suggest this connection, but it could use some stronger connections.

**We have added text to the introduction and discussion to strenghten this connection in light of PET evidence of high concentrations of GABA_A_ receptors in areas of the prefrontal cortex related to emotional processing (page 2, end of 2^nd^ paragraph, and page 9, 4^th^ paragraph of discussion).**

2. The authors present aims related to correlations/associations between measures, yet have a set of analyses related to physiological measures involving higher order cause and effect conclusion that can be made. Results should correspond to the predictions made in the aims section.

**We have again reviewed the aims and results for consistency. The main aim was to investigate effects of benzodiazepines, whereas design factors of the experimental paradigm are assumed to have intended effects, e.g. higher unpleasantness to negative compared to neutral images. Validation of the paradigm was not an aim of this study.**

Method
1. Given that only males were used, the ability to generalize these results is rather limited. Moreover the population of individuals who would take benzodiazepines would likely show the greatest benefit and likely different results for any type of down regulation of emotion as compared to a “normal” sample.

**These important limitations are noted in the discussion (last paragraph).**

2. What was the average valance and arousal for the sets of IAPS images that were used?

**This has been added under Stimuli and Experimental Paradigm.**

3. How long was each picture shown for? How long did the rating scale appear on the screen for? The authors mention they used 2 second time windows, but it isn’t clear if the stimuli were presented for only 2 seconds or longer.

**We have added this information in the methods section (“Stimuli and experimental paradigm”), please see also figure 1.**

Results
a. Much of the results section reported results that were not significant. There were a few interaction effects that were typical and expected.

**We agree with the reviewer’s observation.**

b. The final sample size is reported to be 22, but the table for the placebo condition it appears to say that only 13 participants were in the placebo condition, giving the treatment condition significantly more participants. This could account for a lack of findings in many areas.

**We agree that this is indeed the case, and we discuss this limitation in the discussion section (last paragraph).**

c. Corrugator activity shows expected increases with unpleasant stimuli in the upregulation condition, but no impact it appears of the treatment. Moreover, any of the results that are main effects should only be reported and not interpreted if there is a significant interaction within that set of analyses (e.g. the corrugator activity analyses).

**We have removed interpretations of main effects in the presence of interaction effects throughout the results section.**

d. The majority of the analyses reported appear to have only correlations.

**We agree with the reviewer’s observation.**

Discussion
The analyses that should have resulted in some of the main conclusions about this study appear to be pushed aside as a mere checks and balances set of analyses. The conclusions that are made about the impact of oxazepam appear to impact the subjective emotional experience but not the physiological experience. That is, the results indeed suggest that the participants may interpret that they should feel a particular way without effectively performing the task.

**We agree that effects of oxazepam are evident in self-rated unpleasantness but not in physiological outcomes. The reviewer suggests that this may be due to demand effects. We discuss this possibility in the manuscript (discussion, 2^nd^ paragraph).**

**While we appreciate that the reviewer wishes to further highlight significant effects of the paradigm, this study did not aim to test the validity of the experimental paradigm, which is based on previous research as described in the introduction.**

3. The timecourse of cognitive reappraisal is an important thing to note here. Cognitive reappraisal does not unfold over the course of 2 seconds and the measures that the authors are using rely on autonomic nervous system changes. That is, from an EEG perspective the timecourse of changing brain activity as a result of the cognitive reappraisal task is indeed valid; however, the timecourse to change peripheral physiological responses may differ greatly. This leads back to my question within the method related to how long the stimuli were presented for. It is likely the case that if there was a longer timeframe that the physiological measures were captured there may have been changes late in the stimulus presentation for ECG and skin conductance.

**Effects were measured up to 6 seconds; please see figures 3, 5, and 7.**
